# Antipyrin

**Published:** 1890-01-11

**Authors:** 


### ANTIPYRIN.

The conventional name of antipyrin is probably too firmly established to be discarded, but while its use as a depressor of pyrexia is not always satisfactory or free from risk, its reputation as a nervous sedative is rising continuously. In the treatment of migraine it acts like a charm, as well as in some forms of central or reflex vomiting, and it is of service in pain dependent on certain diseases of the spinal cord. Dr. Archangelsky now reports three cases, diverse enough, though having a common neurotic origin, in which it gave brilliant results. An anæmic and nervous woman who had suffered for four years from distressing urticaria recurring every morning and evening, each attack lasting four hours or so, and extending over her whole body. Iron, arsenic, quinine, bromides, etc., had been tried in vain, but ten-grain doses of antipyrin an hour or two before the time of the expected attacks effected a complete cure in two months, the improvement appearing within the first week. The second case was one of polyuria, in which the urine was reduced in a few weeks from seven to two litres, and no relapse had occurred in six months. The third was severe bronchial asthma, caused by the pressure of a mediastinal tumour on the recurrent nerve. The patient ultimately died, but ten to fifteen grains of antipyrin two or three times a day always afforded relief.

